# Evolutionary Computation with Spatial Receding Horizon Control to Minimize Network Coding Resources

**DOI:** 10.1155/2014/268152

**Published:** 2014-04-14

**Authors:** Xiao-Bing Hu, Mark S. Leeson

**Affiliations:** ^1^State Key Laboratory of Earth Surface Processes and Resource Ecology, Beijing Normal University, Beijing 100875, China; ^2^School of Engineering, University of Warwick, Coventry CV4 7AL, UK

## Abstract

The minimization of network coding resources, such as coding nodes and links, is a challenging task, not only because it is a NP-hard problem, but also because the problem scale is huge; for example, networks in real world may have thousands or even millions of nodes and links. Genetic algorithms (GAs) have a good potential of resolving NP-hard problems like the network coding problem (NCP), but as a population-based algorithm, serious scalability and applicability problems are often confronted when GAs are applied to large- or huge-scale systems. Inspired by the temporal receding horizon control in control engineering, this paper proposes a novel spatial receding horizon control (SRHC) strategy as a network partitioning technology, and then designs an efficient GA to tackle the NCP. Traditional network partitioning methods can be viewed as a special case of the proposed SRHC, that is, one-step-wide SRHC, whilst the method in this paper is a generalized *N*-step-wide SRHC, which can make a better use of global information of network topologies. Besides the SRHC strategy, some useful designs are also reported in this paper. The advantages of the proposed SRHC and GA for the NCP are illustrated by extensive experiments, and they have a good potential of being extended to other large-scale complex problems.

## 1. Introduction

**Figure 1:**
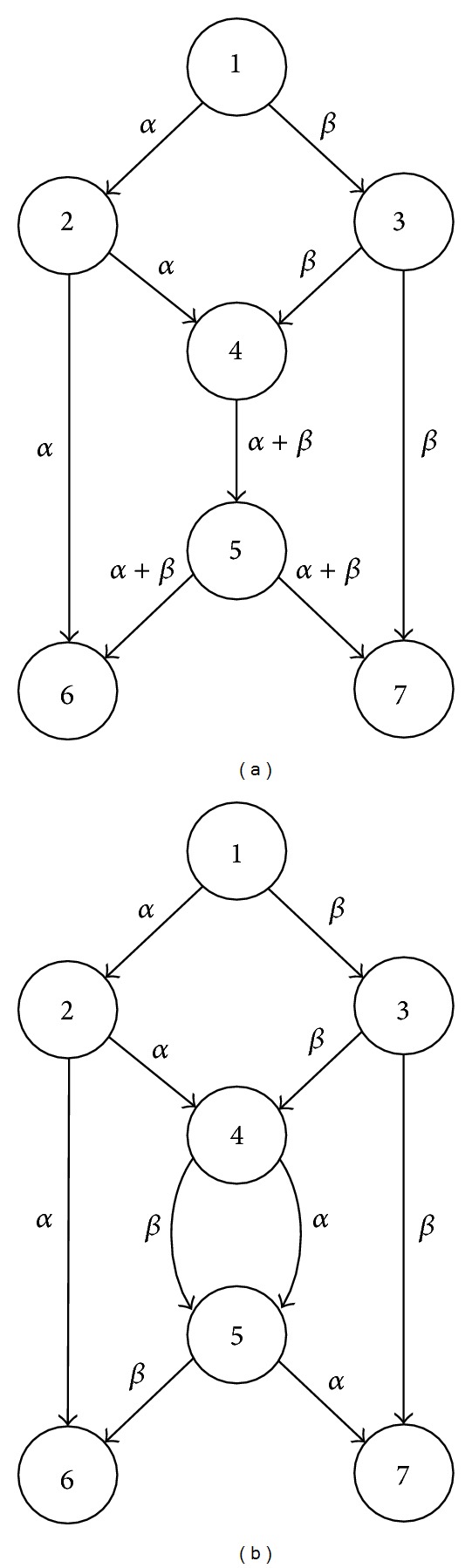
Basic idea of network coding.

Network coding may significantly improve network performance in terms of network throughput [1, 2]. This advantage of network coding is demonstrated in Figure 1(a), where node 1 is the source, nodes 6 and 7 are sinks, and the capacity of every link is just 1 (in this paper, all links are of unit-capacity). If the nodes in the network only forward and replicate the data they receive, then it is easy to see that one sink can only receive 1 unit of data at one time, although the other sink may achieve a rate of 2. However, with network coding allowed, node 4 in Figure 1(a) may combine data from its two incoming links through the “+” operation, and then at both sinks, a rate of 2 can be achieved by using the “−” operation to decode data. Therefore, network coding increases the total rate of information flow through the same network from 3 to 4, which is obviously a significant improvement. Although network coding is usually allowed at all nodes in most relevant literature, an interesting observation is that a given target rate can often be achieved by conducting network coding at only a relatively small proportion of the nodes [2]. For instance, in the network given by Figure 1(b), network coding at both node 4 and node 5 will make no difference in terms of the achieved rate at the sinks. In other words, network coding is not necessary in the network of Figure 1(b). Therefore, a question is raised: at which nodes does network coding need to be conducted, or how to make most of network capacity at a minimal cost in terms of network coding resources? To answer this question, a minimal set of nodes needs to be found for coding, which has been proved to be an NP-hard problem [3].

In this paper, the above problem of minimizing network coding resources is referred to as the network coding problem (NCP). To address this problem, researchers have already attempted many different methods such as minimal approaches [4, 5], linear programming methods [6], and genetic algorithms (GAs) [2, 7–10]. These methods were all reported to be effective to minimize network coding resources. In particular, like in the applications to many other NP-hard problems, GAs as large-scale parallel stochastic searching and optimization algorithms have demonstrated good potential in resolving the NCP. However, the poor scalability of these reported methods largely hampers their applications in the large-scale NCP. For instance, the approaches in both [4, 5] determined the minimal set of nodes for coding by removing links in a greedy fashion. The optimal formulations of the linear programming method in [6] involve a number of variables and constraints that grows exponentially with number of sinks. As a family member of population-based algorithms, GAs are generally very expensive in terms of memory demand and computational time in the case of large-scale problems [11, 12]. To address the scalability problem, decentralized and distributed versions of algorithms often need to be developed, such as the GAs reported in [7, 9]. Before such decentralized and distributed algorithms can be applied, a problem partitioning method has to be employed in order to divide a large-scale network into some subgraphs of manageable size. This paper attempts to shed a bit of more light on how to design an effective scalable GA for the NCP.

In a conventional problem partitioning method (e.g., see [13, 14]), a large-scale problem is divided into some separate subproblems. Then, each subproblem is resolved in a rather isolated manner. After all subproblems have been resolved independently, their subsolutions are integrated together to form a complete solution to the original large-scale problem. However, even though optimal subsolutions to the subproblems can be found, the integrated complete solution to the original large-scale problem is often not optimal or even good. In other words, optimal subsolutions to the subproblems are often not optimal at all from a global point of view. A main cause of losing the global optimality is the independent/isolated way of resolving each subproblem. In this paper, inspired by the temporal receding horizon control (TRHC) strategy in the area of control engineering [15, 16], we propose a novel spatial receding horizon control (SRHC) strategy to partition a large-scale problem. In the SRHC problem partitioning method, a large-scale problem is divided into many subproblems, which compose a problem space; a spatial horizon is then defined which covers some subproblems each time and will recede in the problem space. The spatial horizon is composed of several spatial steps. Each time the spatial horizon recedes by a spatial step. All subproblems covered by a spatial horizon will be optimized as a whole, and only the subsolutions to the subproblems within the first step of the spatial horizon will be saved and fixed, whilst others will be discarded and then recalculated in the next spatial horizon. With the SRHC strategy, a subproblem will be optimized not in an independent/isolated manner, but by making use of its neighboring information in the problem space. Simply speaking, the conventional problem partitioning strategy can be viewed as a one-step-wide SRHC, whilst the new method proposed here is a generalized *N*-step-wide SRHC. Obviously, by optimizing a subproblem together with its neighboring sub-problems, it is likely to improve the quality of the associated subsolution in terms of global optimality. The solution quality may be further improved by integrating a GA into the SRHC method by setting up a solution pool for the subproblems in those decided spatial steps.

Besides the novel SRHC strategy, this paper also integrates some useful designs reported in [10]. There is a common assumption in many studies on the NCP: the target rate is always achievable if coding is allowed at all nodes. To avoid this unrealistic assumption, a more general objective function will be used, which aims not only to minimize network coding resources, but also to maximize the actually achieved rate at sinks. Regarding chromosome structure, rather than the widely used binary matrix, an integer-based permutation representation is adopted, which records relative signals on links and is therefore free of feasibility problems. The permutation representation also enables the derivation of exact information flow on links, which makes it possible to integrate many useful NCP-specific heuristic rules into the algorithm, in order to significantly improve the overall quality of chromosomes. The remainder of this paper will give the details of the proposed SRHC based GA for the NCP.

## 2. Basic Idea of SRHC

### 2.1. Temporal Receding Horizon Control (TRHC) for Dynamic Problems

First of all, a brief review on the conventional receding horizon control (RHC) strategy in control engineering will be very useful. To distinguish from the method proposed in this paper, the conventional RHC in dynamic control problems is hereafter referred to as temporal receding horizon control (TRHC). TRHC, also known as model predictive control, has proved to be a highly effective online optimization strategy in the area of control engineering, and it exhibits many advantages against other control strategies [15, 16]. It is easy for TRHC to handle complex dynamic systems with various constraints. It also naturally exhibits promising robust performance against uncertainties since the online updated information can be sufficiently used to improve the decision. Simply speaking, TRHC is an *N*-step-ahead online optimization strategy to deal with dynamic problems. In this framework, decision is made by looking ahead for *N* steps in terms of a given cost/criterion, and the decision is only implemented by one step. Then the implementation result is checked, and a new decision is made by taking updated information into account and looking ahead for another *N* steps.

**Figure 2:**
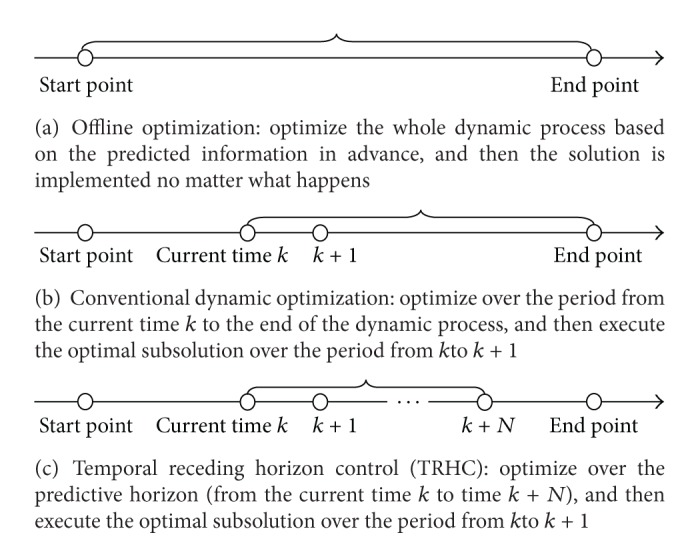
Illustration of temporal receding horizon control (TRHC).

Figure 2 illustrates the basic idea of TRHC by comparing it with some other optimization strategies in an intuitive way. Apparently the offline optimization strategy, as shown in Figure 2(a), is not suitable for dynamic environments. The conventional dynamic optimization, as shown in Figure 2(b), is often criticized for its poor real-time properties and poor performance under disturbances and/or uncertainties in dynamic environments. As illustrated in Figure 2(c), thanks to the idea of temporal receding horizon, the TRHC strategy provides a possible solution to the problems confronted by the conventional dynamic optimization strategy. A properly chosen temporal receding horizon can effectively filter out most unreliable information and reduce the scale of problem. The latter is especially important for complex systems and time-consuming algorithms to satisfy the time limit on the online optimization process. TRHC has now been widely accepted in the area of control engineering [15, 16]. Attention has also been paid to applications of TRHC to areas like management and operations research [17–19]. Particularly, the TRHC strategy has recently been reported to be successfully integrated into population-based algorithms to tackle various dynamic NP-hard optimization problems [20–22].

### 2.2. Spatial Receding Horizon Control (SRHC) for Static Problems

Inspired by the fact that the success of the TRHC strategy largely results from decomposing a complex dynamic process into a serial of temporally associated subprocesses, here we are thinking of how to extend the basic idea of TRHC in order to decompose a large-scale static problem into a serial of associated subproblems (please note that conventional partitioning methods decompose a static problem into a set of separated subproblems). Then in what terms could subproblems be associated in static environments? Basically, we need to create a problem-specific artificial space, project into the space all parts that compose a solution to the original static problem and then design a spatial horizon which recedes in the space. As the spatial horizon recedes out, the value/status of each part will be optimized along together with all other parts that are within the current horizon scope. Once the values/statuses of all parts are optimized, a final solution to the original static problem is determined. Now, one can see that subproblems will be spatially associated in the artificial space. Therefore, hereafter, we call our new strategy for decomposing static problems as spatial receding horizon control (SRHC).

**Figure 3:**
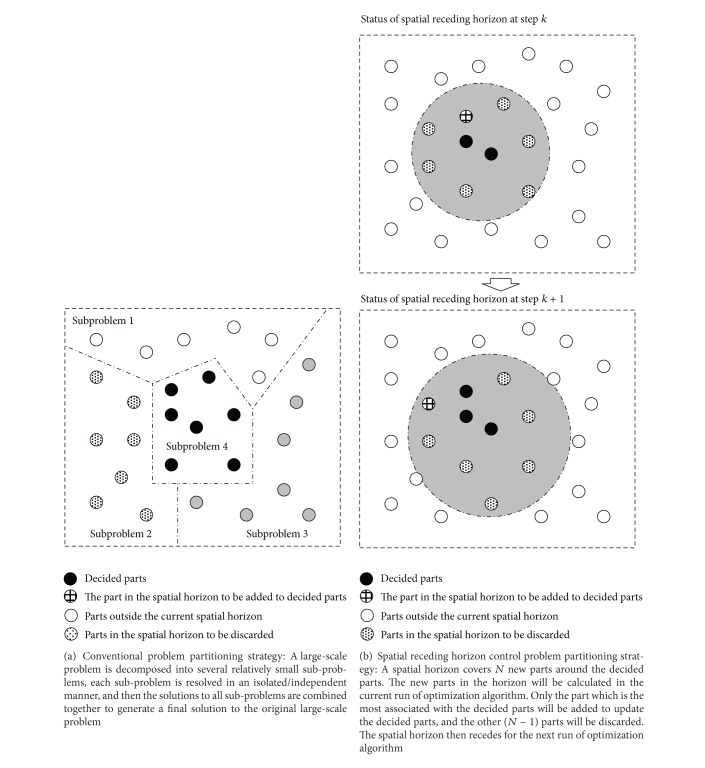
Illustration of Spatial Receding Horizon Control (SRHC).

After an artificial space is designed and all parts that compose a solution are projected into the space, it is crucial to design a spatial horizon receding process to decompose the original static problem into a serial of spatially associated subproblems. A basic spatial horizon receding process can be described as follows. Suppose a solution to a large-scale static problem is composed of *M* local parts. The SRHC strategy makes use of spatial structure (where positions indicate strength of influence between parts of a solution) to move from purely local, part-by-part, optimization to using information from the neighbouring, subglobal context. An optimization algorithm is applied *M* times to determine the *M* parts in a solution. Starting with a specified part, the algorithm calculates at each time step the *N* new parts (usually *N* ≪ *M*), which are the most associated with the* decided* parts, (i.e., parts which have already been optimized in the previous iterations). Although the algorithm will optimize *N* parts each time, only the part that is the most associated with the decided parts will be added to the list of decided parts. The other *N* − 1 parts will be discarded to be recalculated in later iterations. The algorithm keeps running until all *M* parts have been optimized. This leads to a general *N*-step-wide static problem partitioning method, as illustrated in Figure 3. Existing problem-partitioning methods may be considered as a one-step-wide SRHC strategy; that is, each part of a solution is determined in an isolated manner; for example, see [13]. In the generalized *N*-step-wide SRHC strategy, each part is calculated by referring to its most relevant surrounding parts. In other words, subglobal information is used in the determination of a local part. The extra information considered by the *N*-step-wide SRHC strategy can improve the quality of each part and that of the global solution.

Apparently, the design of the artificial space and the spatial horizon receding process is a highly problem-specific task. In this paper, we will particularly discuss how to apply the SRHC strategy to decompose the NCP. After seeing those successful implementations of TRHC based evolutionary algorithms in dynamic environments [20–22], we will also make an attempt to investigate whether integrating SRHC into GAs can deliver a powerful algorithm to resolve the static large-scale NCP.

### 2.3. SRHC and GA: A Perfect Match

**Figure 4:**
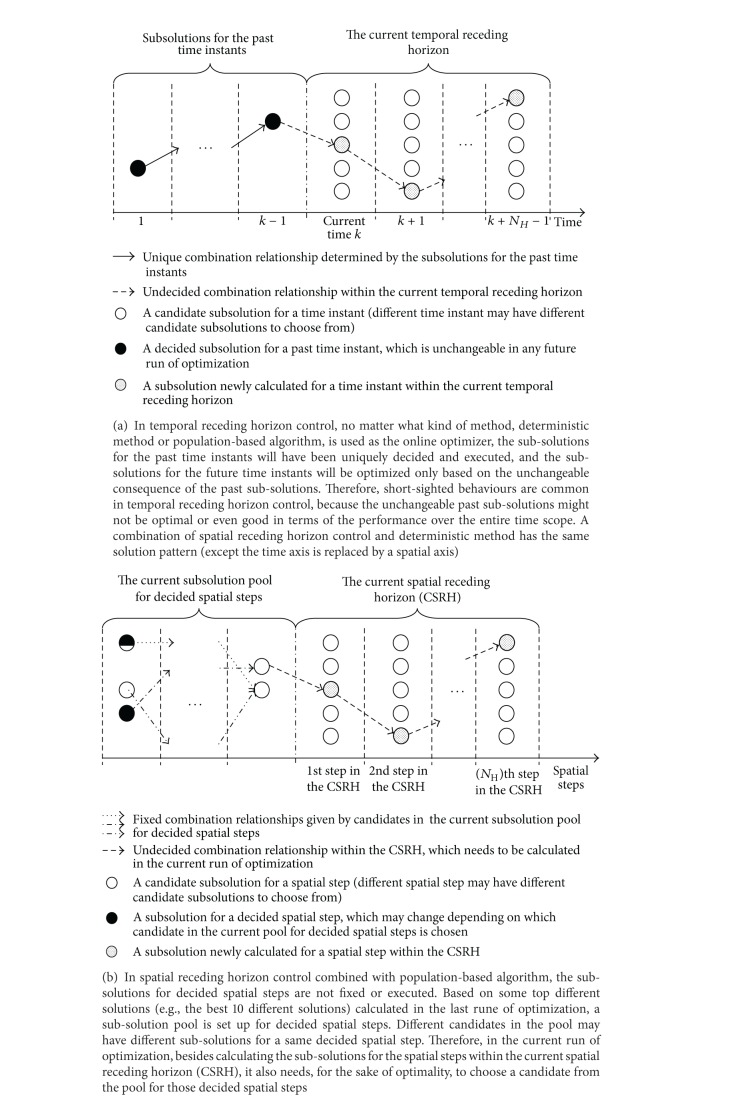
Patterns of sub-solutions in TRHC and SRHC.

**Figure 5:**
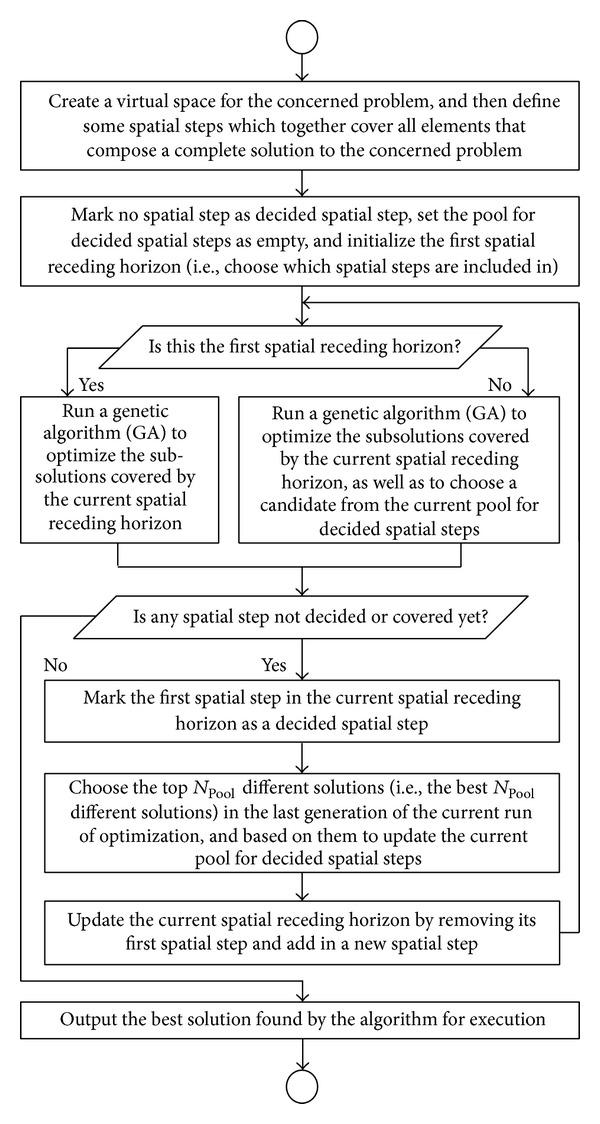
Flowchart of SRHC with GA as online optimizer.

Like the TRHC scheme having an online optimizer, the proposed SRHC also needs to run optimization repeatedly as the spatial horizon recedes step by step. General speaking, any optimization algorithm, deterministic or population-based, can be used by the SRHC strategy as long as it suits the concerned problem. However, in this study, we choose GA, because the SRHC strategy and population-based algorithms like GAs are a naturally perfect match to resolve large-scale static problems. On one side, population-based algorithms are very costly in terms of computational time and resources [23, 24]. Such computational costs often soar up exponentially as the problem scale increases. Therefore, an effective problem decomposing method like the proposed SRHC is crucial for a population-based algorithm to apply to large-scale problems. On the other side, like all other problem partitioning methods, losing global optimality or having shortsighted performance is still an issue the proposed SRHC has to address. If an algorithm, such as a deterministic algorithm, only outputs a single solution, then due to the receding horizon, the subsolutions for decided spatial steps will be uniquely determined and have no chance to change in future runs, as illustrated in Figure 4(a). The uniqueness of the subsolutions for decided spatial steps is a major cause of losing global optimality, because an optimal subsolution calculated within a spatial receding horizon may not be optimal or even good at all from a global point of view. If a population-based algorithm is employed, then the optimization within a spatial receding horizon will generate a population of solutions. Some top solutions in the population may usually have different subsolutions for decided spatial steps. A subsolution pool for decided spatial steps can then be set up according to such top solutions in the population. In the optimization of next spatial receding horizon, it needs not only to calculate those subsolutions covered by the new spatial receding horizon, but also to choose subsolutions from the pool for decided spatial steps. This is illustrated in Figure 4(b). It should be noted that the subsolution pool for decided spatial steps not only records independent subsolutions for each decided spatial step, but more importantly, also records the combination relationships between them as in the associated top solutions. Regarding the decided spatial steps in the new run of optimization, it actually only needs to choose a combination relationship saved in the pool. This can significantly reduce the search space for decided spatial steps. For instance, in Figure 4(b), the independent subsolutions saved in the pool may have at least 6 combinations for decided spatial steps, but the choice needs to be made between only 3 combinations as given by the previous run of optimization. At the same time, the global performance can be effectively improved, because some flexibility in the subsolutions for decided spatial steps is introduced by the pool referring to some top solutions of the previous spatial receding horizon. Therefore, a population-based algorithm like GA can help to improve the global performance of the SRHC.  Figure 5 summarizes the combination of the SRHC scheme with GA as a flowchart.

## 3. Modeling NCP Based on SRHC

### 3.1. Conventional Model of NCP

Suppose a network, denoted as *G*{*V*, *E*} hereafter, where *V* and *E* are sets of vertices and edges, that is, nodes and unit-capacity links in this paper, respectively, has *n*_*n*_ nodes and *n*_*l*_ links. This paper considers only the one-source-multisink SNCP, so, all data originate from a certain node and need to go to some other nodes. For the sake of simplicity, but without losing generality, in this paper it is assumed that the source is always node 1 in the network. Let *n*_*d*_ be the number of signals originating from the source, *n*_*s*_ be the number of sinks in *G*{*V*, *E*}, and *R*_Target_ be the target rate which is expected to be achieved at every sink. Basically, a network protocol and coding scheme define how each node in the network forwards, replicate, and/or encodes data. For instance, assuming that node *i* has *n*_In⁡_(*i*) incoming links and *n*_Out_(*i*) outgoing links and the signal on the *j*th incoming link is *s*_In⁡_(*i*, *j*), then a network protocol and coding scheme can be viewed as a mapping process to generate the signals on outgoing links; that is, *s*_Out_(*i*, *j*),  *i* = 1,…, *n*_*n*_, *j* = 1,…, *n*_Out_(*i*). Mathematically, a network protocol and coding scheme can be denoted as

(1) MNPCS:{G(nn,nl),sIn⁡(1,h)}⟶sOut(i,j)h=1,…,nd, i=1,…,nn, j=1,…,nOut(i).

The most widely used coding operation is linear network coding, which can be mathematically formulated as follows for an outgoing link:

(2) sOut(i,j)=∑h=1nIn⁡(i)w(i,j,h)sIn⁡(i,h),  i=1,…,nn,   j=1,…,nOut(i),

where *w*(*i*, *j*, *h*), *h* = 1,…, *n*_In⁡_(*i*) are weights determining how to combine the *n*_In⁡_(*i*) incoming signals of node *i* to generate a signal for the *j*th outgoing link of node *i*. In theory, *w*(*i*, *j*, *h*) may be continuous, but as proved by [25, 26], sufficient finite discrete values for *w*(*i*, *j*, *h*) can guarantee that the maximum possible throughput is achieved. Therefore, in this paper, *w*(*i*, *j*, *h*) will choose its value from a finite set Θ_*W*_. Assuming that Θ_*W*_ has *N*_*W*_ ≥ 2 discrete values, then the field size for network coding is *N*_*W*_ in this study. A linear coding scheme is actually defined by a set of *w*(*i*, *j*, *h*), in other words, all that is required in order to design a linear coding scheme is the appropriate choice of *w*(*i*, *j*, *h*). Apparently, a network protocol and coding scheme is actually determined by the set of *w*(*i*, *j*, *h*). For a given network protocol and coding scheme, suppose the numbers of coding nodes and links are *N*_CN_ and *N*_CL_, respectively, and the actually achieved rate at sink *i* is *R*(*i*).

With the above preparation, the NCP in this paper is formulated as the following maximization problem:

(3) max⁡MNPCSf1=max⁡w(i,j,h)f1,i=1,…,nn, j=1,…,nOut(i), h=1,…,nIn⁡(i),

where

(4) f1={α1min⁡(R(i))+α2ave(R(i))+α3(NCL+1)+α4(NCN+1), min⁡(R(i))<RTarget,α1min⁡(R(i))+α2ave(R(i))+α5(NCL+1)+α6(NCN+1), min⁡(R(i))≥RTarget,i=1,…,ns

*α*_*k*_, *k* = 1,…6, are weights, and

(5) $$\begin{array}{c} \min\left(\alpha_{1},\alpha_{2}\right)>\max\left(\alpha_{3},\alpha_{4}\right),\\ \min\left(\alpha_{5},\alpha_{6}\right)\gg \max\left(\alpha_{1},\alpha_{2}\right), \end{array}$$

subject to *G*(*n*_*n*_, *n*_*l*_). Clearly, this maximization problem aims to find a network protocol and coding scheme to maximize *f*_1_ defined by (4) and (5). From the above objective function, one can see that the NCP will firstly try to maximize the overall actually achieved rate, and once the target rate is achieved, the focus of the optimization will switch to minimizing the network coding resources. The term “min⁡(*R*(*i*))” and term “ave(*R*(*i*))” in (4) can be used to assess the actually achieved rate. Basically, a larger term value for “ave(*R*(*i*))” is desirable. *R*(*i*) should be optimized as evenly as possible; that is, increasing the rate at some sinks by largely sacrificing the rate at other sinks should be avoided. This can be reflected by the term value for “min⁡(*R*(*i*));” that is, the larger the value is, the more evenly *R*(*i*) is optimized. At the same time, as reflected by the term “1/(*N*_CL_ + 1)” and the term “1/(*N*_CN_ + 1)  ,” the network coding resources should be minimized, particularly when the target rate can be achieved, that is, when min⁡(*R*(*i*)) ≥ *R*_Target_.

### 3.2. SRHC Based Model for NCP

To design an SRHC based model for the NCP, firstly we need to create an artificial space, then to project all network nodes into the space, then to design a spatial horizon receding process, and at last to reformulate the maximization problem given by (3) to (5) in order to make it fit in the SRHC framework.

Usually, a network where coding needs to be performed already defines its own space (real or virtual) and may have its nodes distributed in the space in a rather random manner, but such a space and the distribution of nodes are of little use to the design of artificial space and the projection of nodes in the SRHC model for the NCP. In the SRHC model, we simply use a purely imaginary two-dimensional space and then project network nodes into the space according to the connections between nodes. The projecting procedure is described as follows.

**Step 1**

Let *M*_LN_(*i*) be the set that records all nodes in the *i*th node layer, and *M*_LL_(*i*) records all links in the *i*th link layer. Start from the source, that is, node 1. Set node 1 as the only node in *M*_LN_(1), and set the end nodes of all outgoing links of node 1 as the nodes in *M*_LN_(2). Then set the current layer *l*_*C*_ = 2. Let Ω_*N*_ be the set of all nodes that are not included in *M*_LN_(1) and *M*_LN_(2).

**Step 2**

While Ω_*N*_ ≠ *Ø*, do

*Substep 2.1*. Put all end nodes of all outgoing links of the nodes in *M*_LN_(*l*_*C*_) as the nodes in *M*_LN_(*l*_*C*_ + 1).

*Substep 2.2*. If a node in *M*_LN_(*l*_*C*_ + 1) is already included in *M*_LN_(*i*), *i* = 1,…, *l*_*C*_, then remove this node from *M*_LN_(*i*), and add it to *Ω*_*N*_.

*Substep 2.3*. Remove all nodes of *M*_LN_(*l*_*C*_ + 1) from Ω_*N*_. Let *l*_*C*_ = *l*_*C*_ + 1.

**Step 3**

Create a two-dimensional space, where the *x* axis is the node layer number, and the *y* axis has no specific meaning. Then project all nodes into the space according to *M*_LN_. For instance, suppose a node belongs to *M*_LN_(*i*). Then the *x* coordinate of this node is *i*. The *y* coordinate of this node can be random, but for distinguishing purposes, the nodes in the same layer should be assigned with different values of *y*.

**Step 4**

For a link, suppose its starting node is within *M*_LN_(*i*) and its end node within *M*_LN_(*i*), *j* > *i*. Then add this link to *M*_LL_(*i*),…, *M*_LL_(*j* − 1).

**Figure 6:**
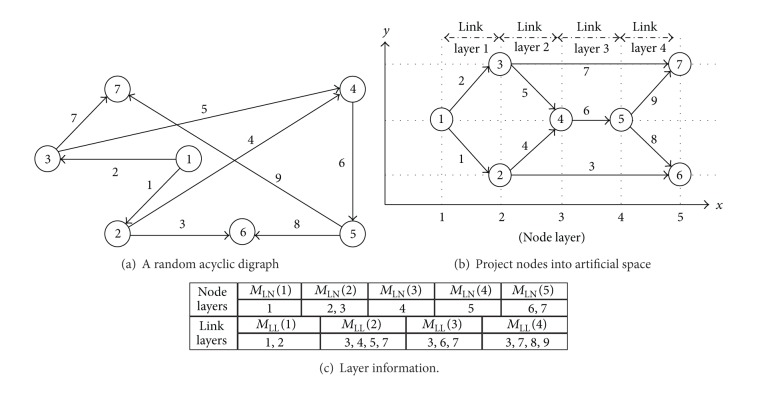
An illustration of node projecting procedure.

**Figure 7:**
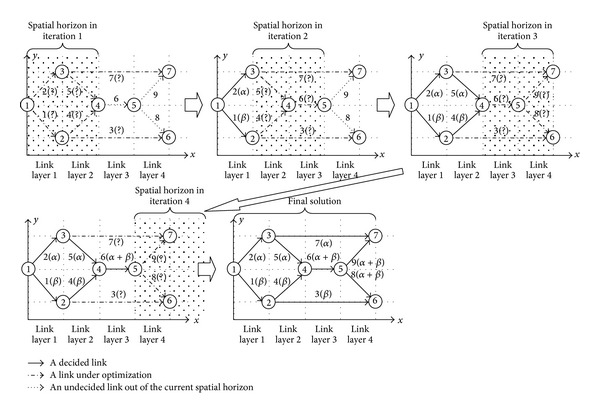
An illustration of spatial horizon receding process.

Figure 6 gives a simple illustration about the above node projecting procedure. The information of node layers and link layers is crucial not only to define the spatial horizon, but also to design the spatial horizon receding process for the NCP. As illustrated in Figure 7, the spatial horizon for the NCP is defined based on link layers. In each iteration of optimization, the spatial horizon covers some successive link layers; for example, in the case of Figure 7, the spatial horizon spans over two successive link layers. In an iteration of optimization, only those links that are covered by the current spatial horizon will be optimized. The spatial horizon recedes for one link layer each time along the *x* axis in the artificial space. In the new iteration of optimization, those links that have been optimized in the previous iteration of optimization and get out of the current spatial horizon due to the horizon receding process will be fixed as decided links, whilst those links that have been optimized in the previous iteration of optimization but are still within the current spatial horizon will be optimized again along with the links that are newly covered by the spatial horizon. This process continues until all links become decided links. The details of the spatial horizon receding process are given as follows.

**Step 1**

Set up *N*_*H*_, the length of the spatial horizon. Let Ω_*S*_ be the set of sinks, Ω_DL_ = *Ø* the set of decided links, and Ω_UL_ = {*M*_LL_(1),…, *M*_LL_(*N*_LL_)} the undecided links, where *N*_LL_ is the number of total link layers. Let *k* = 1.

**Step 2**

While Ω_UL_ ≠ *Ø*, do

*Substep 2.1*. Set the current spatial horizon as Ω_*H*_(*k*) = {*M*_LL_(*k*),…, *M*_LL_(*k* + *N*_*H*_ − 1)}. It should be noted that *M*_LL_(*i*) = *Ø* for *i* > *N*_LL_.

*Substep 2.2*. Let *W*(*k*) be the weights for the links in Ω_*H*_(*k*), *M*_EN_(*k* + *N*_*H*_ − 1) the set of all end nodes of the links in *M*_LL_(*k* + *N*_*H*_ − 1), and *N*_CL_(*k*) and *N*_CN_(*k*) are the current numbers of coding links and coding nodes, respectively. Then calculate the following maximization problem:

(6) $$\begin{array}{c} \underset{W\left(k\right)}{\max}f_{2}\left(k\right), \end{array}$$

subject to the signals on Ω_DL_, where *f*_2_(*k*) is a new objective function defined as follows:

(7) f2(k)=β1fNT(MEN(k+NH−1))+β2fMR(MEN(k+NH−1))+β3fSD(MEN(k+NH−1))+β4(NCL(k)+1)+β5(NCN(k)+1)+β6fTP(k,ΩS),

which will be explained later.

*Substep 2.3*. Remove *M*_LL_(*k*) from Ω_UL_ to Ω_DL_; that is, Ω_UL_ = Ω_UL_ − *M*_LL_(*k*) and Ω_DL_ = Ω_DL_ + *M*_LL_(*k*). Let *k* = *k* + 1.

**Figure 8:**
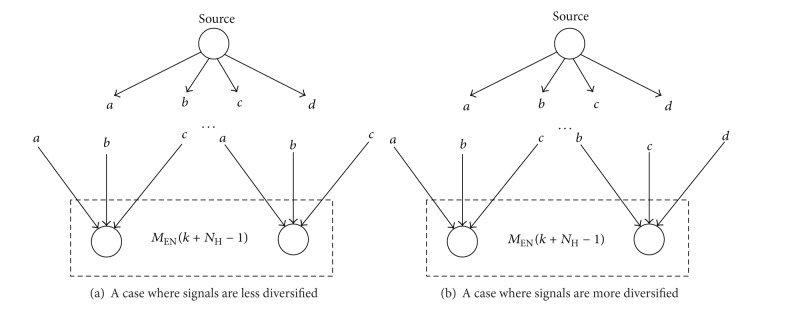
An illustration of signal diversification.

**Figure 9:**
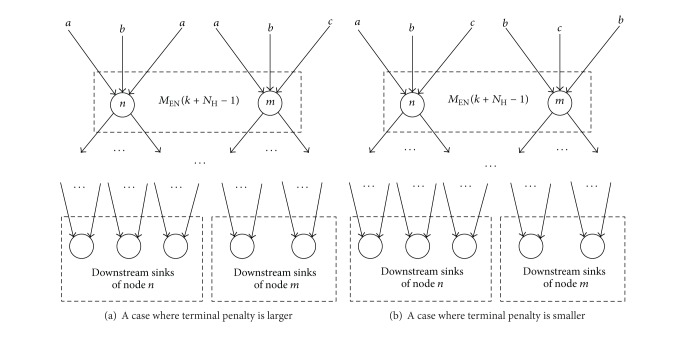
An illustration of terminal penalty.

In the above spatial horizon receding process, a new maximization problem as defined by (6) and (7) needs to be resolved during each iteration of optimization. In the new objective function *f*_2_(*k*), the term *f*_NT_(*M*_EN_(*k* + *N*_*H*_ − 1)) is a function that calculates the network throughput at all nodes in *M*_EN_(*k* + *N*_*H*_ − 1), the term *f*_MR_(*M*_EN_(*k* + *N*_*H*_ − 1)) is a function that calculates the minimal rate over the nodes in *M*_EN_(*k* + *N*_*H*_ − 1), the term *f*_SD_(*M*_EN_(*k* + *N*_*H*_ − 1)) is a function that assesses how much the signals on *M*_EN_(*k* + *N*_*H*_ − 1) are diversified, the term *f*_TP_(*k*, Ω_*S*_) is a terminal penalty which assesses the impact of the current stage solution on the network throughput at the sinks, and *β*_1_ to *β*_6_ are weights to combine different terms. Apparently *f*_2_(*k*) is quite different from the objective function of the conventional NCP model, that is, *f*_1_ as defined in (4), mainly because of two new terms: *f*_SD_(*M*_EN_(*k* + *N*_*H*_ − 1)) and *f*_TP_(*k*, Ω_*S*_). The reason for introducing *f*_SD_(*M*_EN_(*k* + *N*_*H*_ − 1)) is illustrated in Figure 8, where one can see that, assuming all other terms in *f*_2_(*k*) are the same, Figure 8(a) is better than Figure 8(b) because the signals on *M*_EN_(*k* + *N*_*H*_ − 1) are better diversified, which means the downstream nodes will have more choices. The introduction of *f*_TP_(*k*, Ω_*S*_) is in line with the common practice of TRHC in the area of control engineering, which aims to minimize shortsighted behaviors, such as getting trapped in local optima and generating unstable/unconverged solutions, due to the fact that not all information is covered by the receding horizon. Before running the SRHC model, we need to count the number of downstream sinks for every node in the network. Then we can roughly assess the final impact of the signals received by a node. Basically, a node with more downstream sinks deserves a higher priority to receive more signals, just like Figure 9 illustrates.

The absolute value of different term in *J*_2_ may vary in quite different range; for example, *f*_NT_ may be over 1000 whilst *f*_MR_ may be smaller than 10. This means the absolute values of different terms in *J*_2_ are usually incomparable, and then they cannot be directly combined by *J*_2_. Therefore we need to unify the terms of *J*_2_; in other words, we have to use the relative value of each term, as defined by the following:

(8) f3(k)=β1f−NT(MEN(k+NH−1))+β2f−MR(MEN(k+NH−1))+β3f−SD(MEN(k+NH−1))+β4(NCL(k)+1)+β5(NCN(k)+1)+β6f−TP(k,ΩS),

(9) $$\begin{array}{c} {\overset{-}{f}}_{\text{NT}}\left(M_{\text{EN}}\left(k+N_{H}-1\right)\right)=\frac{f_{\text{NT}}\left(M_{\text{EN}}\left(k+N_{H}-1\right)\right)}{F_{\text{NT}}\left(M_{\text{EN}}\left(k+N_{H}-1\right)\right)}, \end{array}$$

(10) $$\begin{array}{c} {\overset{-}{f}}_{\text{MR}}\left(M_{\text{EN}}\left(k+N_{H}-1\right)\right)=\frac{f_{\text{MR}}\left(M_{\text{EN}}\left(k+N_{H}-1\right)\right)}{F_{\text{MR}}\left(M_{\text{EN}}\left(k+N_{H}-1\right)\right)}, \end{array}$$

(11) $$\begin{array}{c} {\overset{-}{f}}_{\text{SD}}\left(M_{EN}\left(k+N_{H}-1\right)\right)=\frac{f_{\text{SD}}\left(M_{\text{EN}}\left(k+N_{H}-1\right)\right)}{F_{SD}\left(M_{EN}\left(k+N_{H}-1\right)\right)}, \end{array}$$

(12) $$\begin{array}{c} {\overset{-}{f}}_{\text{TP}}\left(k,\Omega_{S}\right)=\frac{f_{\text{TP}}\left(k,\Omega_{S}\right)}{F_{\text{TP}}\left(k,\Omega_{S}\right)}, \end{array}$$

where *F* is a function to calculate the value of a term in an ideal condition; for example, *F*_NT_ and *F*_MR_ assume that every node in *M*_EN_(*k* + *N*_*H*_ − 1) receives as many different signals as its incoming links or all signals sent from the source, whichever is larger, *F*_SD_ assumes that *M*_EN_(*k* + *N*_*H*_ − 1) receives as many different signals as the links in *M*_LL_(*k* + *N*_*H*_ − 1) or all signals sent from the source, whichever is larger, and *F*_TP_ assumes all signals received by a node in *M*_EN_(*k* + *N*_*H*_ − 1) can be sent to its every downstream sink. Apparently, the value of a unified term, that is, $\overset{-}{f}$, is within [0 1], and this makes *f*_3_ more reasonable and easier to tune. The design details of $\overset{-}{f}$ and *F* may vary, and due to limited space, here we skip them.

## 4. SRHC Based GA for NCP

The design of GAs usually includes choosing an appropriate chromosome structure, developing effective evolutionary operators, introducing useful problem-specific heuristic rules, and adjusting algorithm-related parameters. This section will explain the first three aspects, and the last aspect will be discussed in the experiment section. Here we will firstly spend three subsections to describe some useful GA-related designs reported in [10]. Then some SRHC-related modifications to the GA designs will be discussed in order to properly integrate the GA into the SRHC method for the NCP.

### 4.1. Chromosome Structure

The chromosome structures of the GAs in [2, 7, 8] are based on the use of a binary matrix to record the active states of links, and such structures make it easy to apply graph theoretic methods to ascertain whether the target rate is or is not achievable by a given chromosome. A chromosome in [2, 7, 8] does not have full information concerning a specific network protocol and coding scheme and may associate with different specific network protocols and coding schemes. Although this means to some extent random linear coding can be employed, it may be difficult to determine the exact information flow on links. The lack of exact information flow on links will make it difficult not only to calculate the actually achieved rate at sinks, but also to integrate useful NCP-specific heuristic rules into the algorithms. Therefore, in this paper we construct chromosomes based on a permutation representation, in order to record exact information flow on links.

A permutation representation is often used when GAs are being applied to combinatorial problems (actually, the NCP is a combinatorial problem), because it can usually construct chromosomes straightforwardly based on their physical meanings. However, such a representation is often confronted by feasibility problems; that is, a chromosome may become infeasible in terms of its physical meaning during evolutionary operations. Sometimes some evolutionary operators have to be modified significantly or even discarded in order to resolve such problems. For the NCP, a straightforward permutation representation is to use the absolute information flow on links to construct chromosomes, but this representation will cause serious feasibility problems during evolutionary operations, because the set of feasible signals from which a link can choose cannot be predetermined and varies over time according to the signals on other links. This means that any change in the signal on a link caused by evolutionary operations could make the unchanged signals on some other links infeasible.

**Figure 10:**
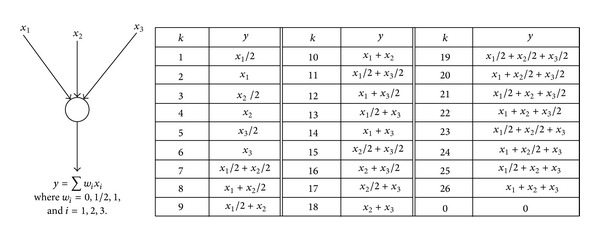
An illustration of definition of signal combinations.

**Figure 11:**
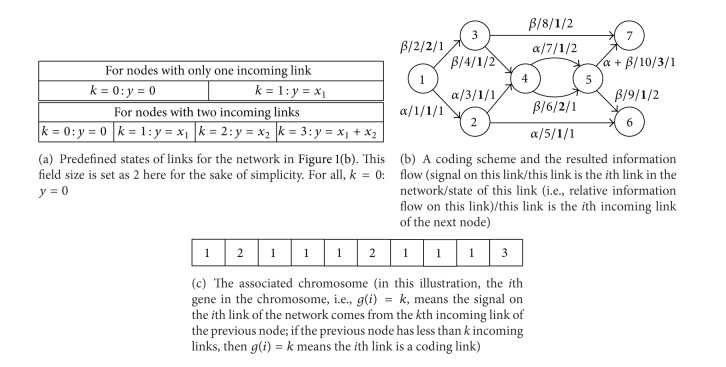
Chromosome structure based on relative information flow on links.

Fortunately, the permutation representation in [10] is free of feasibility problems without sacrificing any of the merits of permutation representations. Instead of the absolute information flow on links, a chromosome in [10] records the relative information flow, that is, an integer *k*, whose meaning is a certain predefined combination of signals on incoming links of a node. For instance, Figure 10 shows an illustration of how to predefine *k*. In Figure 10, a table is set up to define all possible signal combinations at a node with three incoming links, and the field size is *N*_*W*_ = 3. A different number of incoming links require a different predefined table for *k*, as illustrated in Figure 11.

Let *head*(*i*) denote the serial number of the starting node of link *i*. It is assumed that the source has as many incoming links as there are signals to be sent, and each signal is associated with one and only one of such assumed links. Let gene *i*, that is, *g*(*i*), be associated with link *i*.Then*g*(*i*) = *k*, *k* = 0,1,…, *N*_*W*_^*n*_In⁡_(*head*(*i*))^, where *n*_In⁡_(*head*(*i*)) is the number of incoming links from node *head*(*i*). In other words, for an outgoing link, for example, link *i*, the number of possible combinations (including no coding) is *N*_*W*_^*n*_In⁡_(*head*(*i*))^. The exact combination that a value of *k* stands for needs be predefined. Hereafter, the value of *g*(*i*) is called the* state* of link *i*. Then the set of possible* states* for link *i* is

(13) $$\begin{array}{c} \Theta_{S}\left(i\right)=\left\{0,1,\ldots ,N_{W}^{n_{\mathrm{In}}(head(i))}\right\}. \end{array}$$

Therefore, the size of the solution space of the GA is

(14) $$\begin{array}{c} n_{\text{SP}}=\overset{n_{l}}{\underset{i=1}{\prod }}N_{W}^{n_{\mathrm{In}}(head(i))}. \end{array}$$

Unlike the absolute information flow on the links, Θ_*S*_(*i*) only depends on the network topology and the number of signals that are to be sent, which are both fixed during a GA run. Therefore, as long as *g*(*i*) remains within Θ_*S*_(*i*) during the evolutionary operation, there will be no feasibility problem. As will be discussed in the following subsection, this condition is very easily fulfilled. On the other hand, the absolute information flow on links can be derived in a straightforward way from a chromosome of the new GA. The simple illustration in Figure 11 indicates how to use relative information flow on links to construct a chromosome.

**Figure 12:**
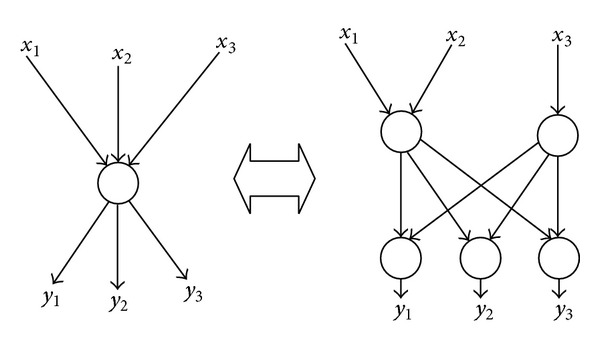
Transform a network into a new network with max⁡(*n*_In⁡_(*i*)) = 2.

The chromosome described above is a vector with a size of *n*_*l*_. One may also use a *n*_*l*_ × max⁡(*n*_In⁡_(*i*)) matrix to record, for each link, the weights applied to its incoming links. Such a matrix representation will need no predefined tables. In this study, we choose the vector representation because (i) it has a lower memory demand, particularly in the case of large-scale networks,and (ii) it is more efficient in terms of algorithm execution (the matrix representation requires to generate *n*_In⁡_(*head*(*i*)) random numbers to determine the relative information flow on link *i*, whilst the vector representation needs only one random number). However, for networks where a node may have many incoming links, the predefined tables for the vector representation will become enormously huge if the field size is also large. For instance, assuming max⁡(*n*_In⁡_(*i*)) = 10 and *N*_*W*_ = 10, then the largest predefined table will have 10^10^ entries for *y*. In this case, we can transform the network into an equivalent network which has a relatively small max⁡(*n*_In⁡_(*i*)). Actually, we can always transform a network into a new one with max⁡(*n*_In⁡_(*i*)) = 2, as illustrated in Figure 12, and then even if *N*_*W*_ = 100, the largest predefined table only needs 100^2^ = 10^4^ entries for *y*. The transformed network will have more links than the original network, which means, according to (14), that the entire search space will increase, which will particularly become of concern in the case of large-scale networks if no problem partitioning method is used. Fortunately, with the proposed SRHC method to decompose large-scale networks, the search space during a spatial receding horizon can easily be restricted to a manageable size, no matter how large the original network scale is.

It should be noted that the search space given by (13) and (14) is much larger than those in previous studies. For instance, the search space size for link *i* is 2^*n*_In⁡_(*head*(*i*))^ in [2], and even down to *n*_In⁡_(*head*(*i*)) + 2 in [7, 8]. Fortunately, this disadvantage can be compensated by introducing some useful problem-specific heuristic rules based on exact information flow on links. Such heuristic rules are difficult to apply to previous chromosome structures such as in the case of [7, 8], because exact information flow on links can hardly be derived there. For instance, the modified binary representation in [7] is even “at the price of losing the information on the partially active link states that may serve as intermediate steps toward an uncoded transmission state.” Differently, the new GA reported in this study can derive the exact information flow on links associated with a chromosome and therefore can take advantage of many useful NCP-specific heuristic rules. As a result, the GA reported here may still find theoretically optimal solutions, despite the huge search space.

### 4.2. Evolutionary Operators

The mutation operator in this paper is designed as follows. A chromosome is chosen for mutation with probability *p*_*m*_. Then a gene associated with a potential coding link needs to be chosen randomly. Suppose the *i*th gene, that is, *g*(*i*), is chosen, whose associated link is the *i*th link in the network. Then the set of possible states for link *i* is given by Θ_*S*_(*i*) as defined in (13). Mutation will randomly choose a value from the set Θ_*S*_(*i*)−{*g*(*i*)} and then reset *g*(*i*) to the new value. Since Θ_*S*_(*i*) only depends on the network topology, the above mutation operation is free of feasibility problems.

This paper adopts uniform crossover, which is highly efficient in not only identifying, inheriting, and protecting common genes, but also in terms of recombining noncommon genes [17, 27]. Simply speaking, in uniform crossover, each gene of an offspring chromosome inherits the associated gene from its two parent chromosomes with a 50% chance. Thanks to the permutation representation, the *i*th genes of all chromosomes share the same set of possible states for link *i*, and, therefore, uniform crossover will cause no feasibility problems. Regarding the choice of two parent chromosomes, any chromosome in an old generation may be chosen as the first parent chromosome at a fixed probability of *p*_*c*_, and then a different chromosome may be chosen as the second parent chromosome at a probability proportional to its fitness. In this way, every chromosome stands the same chance to become the first parent, while a fitter chromosome stands a better chance to cross over with most other chromosomes.

### 4.3. Heuristic Rules

It is well known that heuristic rules, particularly problem-specific rules, often play an important role in successful applications of GAs. What kinds of rules to introduce and how to integrate them into algorithms effectively are challenging tasks and usually need to be taken into account in the GA design stage. The permutation representation discussed in Section 4.1 makes it very easy to integrate the following NCP-specific rules.

**Rule 1**

All evolutionary operations only apply to potential coding nodes and links.

**Rule 2**

When initializing the first generation, a certain proportion of chromosomes will allow coding on all potential coding nodes, and for a potential coding node which has multiple outgoing links, choose at least one link randomly as a coding link. This rule can help to find a solution to achieve the target rate, if it is achievable, at all sinks.

**Rule 3**

Furthermore, in the initialization of the first generation, another proportion of chromosomes will allow no coding at all. This rule can help to explore the possibility of maximizing the rate actually achieved at the minimum cost of resources.

**Rule 4**

In either initialization or evolutionary operations, the states of incoming links of a potential coding node should be determined in such a way that the node will receive as many different signals as possible. In other words, the signals to a potential coding node should be diversified as much as possible. This rule will allow as many choices as possible for network protocols and coding schemes and therefore can help to diversify a generation. It should be noted that it is the proposed permutation representation that makes it possible to integrate this rule into the algorithm, because the information flow on links associated with a chromosome can be easily checked out to see whether the signals to potential coding nodes are effectively diversified.

**Rule 5**

For a potential coding node with multiple outgoing links, there should be a high probability that the outgoing links have different states. This rule also takes advantage of the proposed permutation representation and can help to diversify a generation.

It should be noted that Rules 4 and 5 cannot be used by the methods reported in [2, 7–9], because the application of Rules 4 and 5 demands the availability of the exact information flow on links, which is guaranteed by the chromosome structure adopted in this paper. As will be revealed by the simulation results, Rules 4 and 5 can significantly improve the quality of chromosomes.

### 4.4. SRHC Related Modifications in GA

In order to properly integrate the above GA-related designs into the SRHC for the NCP, two modifications are necessary. One is related to the chromosome structure, and the other to the mutation operation. Hereafter for distinguishing purposes, a GA designed according to the above three subsections is referred to as GlobalGA, because no problem partitioning method is used, whilst a GA with the SRHC-related modifications discussed in this subsection is called SRHCGA. The SRHCGA can also be interpreted as the combination of the SRHC with GA, or an SRHC method with GA as optimizer.

**Figure 13:**
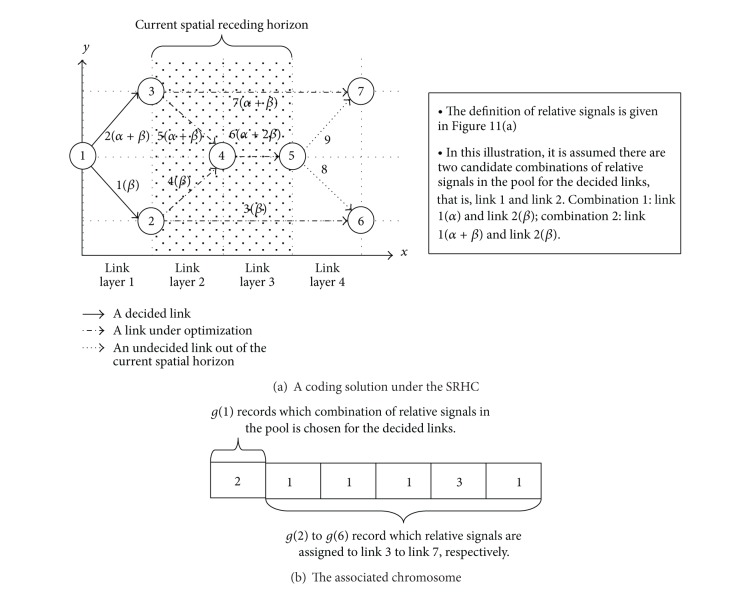
An illustration of the modified chromosome structure for SRHC.

As discussed in Section 2, in the SRHCGA, the optimization within a spatial receding horizon not only needs to calculate the subsolutions to the subproblems covered by the spatial receding horizon, but also has to choose the subsolutions for the subproblems in decided spatial steps. In the case of applying the SRHCGA to the NCP, besides assigning relative signals to the undecided links covered by the current spatial receding horizon, the optimization within the horizon will also choose a combination of relative signals for all decided links, and all candidate combinations are saved in a pool which is updated as the spatial horizon recedes. To be able to make such a choice for decided links, we need an additional special gene in the chromosome structure to record which candidate combination in the pool is chosen. This can be easily done by modifying the chromosome structure in Section 4.1 as follows. Suppose there are *N*_SP_ candidate combinations in the current pool for decided links and *N*_ULSRHC_ undecided links in the current spatial receding horizon. Then the modified chromosome structure has (*N*_ULSRHC_ + 1) genes in total. The first gene records which candidate combination in the pool is chosen for decided links, that is, *g*(1) = *m*, *m* ∈ {1,…, *N*_SP_}, means that the *m*th candidate combination saved in the pool has been chosen to set up the relative signals on decide links. The following *N*_ULSRHC_ genes, that is, *g*(*i*), *i* = 1,…, *N*_ULSRHC_, record the relative signals assigned to the undecided links covered by the spatial horizon, and their definition is exactly the same as described in Section 4.1. All candidate combinations in the pool for decided links are predetermined by the optimization of the previous spatial receding horizon and then are fixed during the optimization of the current spatial horizon. Therefore, the new *g*(1) will cause no feasibility problem, just like other genes which record relative signals.  Figure 13 gives an illustration of the modified chromosome structure for SRHC.

The second SRHC-related modification is made to the mutation operation given in Section 4.2. Actually, the modification is minor: the search space for the new *g*(1) is not given by (13), and *g*(1) always mutates within {1,…, *N*_SP_} − *g*(1).

## 5. Experimental Results

In this section, we will firstly test whether the GlobalGA, which employs no problem partitioning method, is effective to resolve the NCP. Then, we will investigate the performance of the proposed SRHC scheme by comparing the SRHCGA with the GlobalGA. There are two sets of networks, see Table 1 for details, which are taken from [2] for comparative purposes. The networks in Set I are actually generated by the algorithm in [28], which constructs connected acyclic directed graphs uniformly at random. There are two networks used for simulations in Set I: one network, denoted as Case I-1, has 20 nodes, 80 links, 12 sinks, and rate 4, and the other network, denoted as Case I-2, has 40 nodes, 120 links, 12 sinks, and rate 3. The networks in Set II are constructed by cascading a number of copies of network (b) in Figure 1 such that the source of each subsequent copy of network (b) in Figure 1 is replaced with an earlier copy's sink. Set I has 4 networks, which uses fixed-depth binary trees containing 3, 7, 15, and 31 copies of network (b) in Figure 1, respectively. These 4 networks in Set II are referred to as Case II-1 to Case II-4 in this section. These 4 networks have a maximum multicast rate of 2, which is achievable without coding; that is, the optimal solutions have no coding links.

### 5.1. Tests on GlobalGA

Here the GlobalGA developed in this paper will be tested by comparing it with the GA reported in [2] (denoted as GA[2]) and two minimal approaches reported in [4, 5] (denoted as Minimal 1 and Minimal 2, resp.). As discussed in Section 4, the permutation representation makes it very easy to integrate many problem-specific heuristic rules, that is, Rule 1 to Rule 5 given in Section 4.3, which are expected to improve the performance of the GlobalGA. In order to examine whether such heuristic rules really work, three versions of the GlobalGA are used in the experiments: the first version, denoted as GlobalGA1, only employs Rules 1 and 2, the second version, denoted as GlobalGA2, uses one more rule, that is, Rule 3, than GlobalGA1, and the third version, denoted as GlobalGA3, adopts Rule 1 to Rule 5. The reason why GlobalGA1 is included is because it employs exactly the same heuristic rules as used in GA[2]]; therefore, any difference in performance between GlobalGA1 and GA[2] should mainly result from the basic designs, for example, chromosome structures and associated operations, used in [2] and those used in this paper. The reason for including GlobalGA2 is because there is no difficulty in applying Rule 3 to GA[2]. Since Rule 3 can improve the performance of GlobalGA2, one can expect that Rule 3, once applied, might also benefit GA[2]. As mentioned in Section 4, it is because of the permutation representation that the integration of Rules 4 and 5 becomes possible; therefore, GlobalGA3 will reveal the extent to which the GlobalGA reported in this paper is advantageous.

To make a fair comparison, GlobalGA1 to GlobalGA3 have the same population size (150) and upper bound (300) for the number of generations for evolution as GA[2] does. Then 20 random runs of each algorithm are conducted, and the average results are listed in Tables 2 and 3 reveal more details about the performance of GlobalGA1 to GlobalGA3. For the sake of simplicity, in most parts of the simulation *w*(*i*, *j*, *h*) = 0 or 1 in (2), that is, the field size *N*_*W*_ = 2, unless specified otherwise. From these results one can make the following observations.

- Table 2 shows that, in the cases of Set I, that is, Case I-1 and Case I-2, all the methods perform similarly. In more precise terms, the GlobalGAs reported in this paper, that is, GlobalGA1, GlobalGA2, and GlobalGA3, return slightly lower average numbers of coding links than the existing methods. However, since all methods can find the optimal (i.e., no coding required), or almost optimal solutions to both the cases in Set I, we cannot claim that our algorithm has a significant advantage compared with existing algorithms. Analysis of the network topologies in Set I suggests that these networks have too many links; for one network, *n*_*l*_ = 4*n*_*n*_, and for the other, *n*_*l*_ = 3*n*_*n*_. In the Graph Drawing Community, graphs (i.e., networks) having *n*_*l*_ = 4*n*_*n*_ links are actually considered to be dense [28]. In such a network with dense links, it is easy to achieve a relatively small target rate without network coding. Compared with Set I, all the networks in Set II have *n*_*l*_ < 2*n*_*n*_. Therefore, although the target rates in Set II are smaller than those in Set I, it is probably more difficult to find a no-coding solution to achieve the smaller target rates in Set II. Actually, in the Set II cases, that is, Case II-1 to Case II-4, the results of a comparison of these methods show significant differences, which may suggest that the networks in Set II are more suitable for testing different methods. Therefore, hereafter, we will only focus on analyzing the results of Case II-1 to Case II-4.
- Table 2 also shows that, in Case II-1 to Case II-4, GlobalGA1, GlobalGA2, and GlobalGA3 clearly outperform the existing algorithms, that is, Minimal 1, Minimal 2, and GA[2]. Unlike the existing algorithms, which can hardly find the theoretically optimal solutions, particularly in complicated cases such as Case II-3 and Case II-4, all three new GlobalGAs are capable of finding the theoretically optimal solutions in all 4 cases of Set II.
- GlobalGA1 adopts exactly the same heuristic rules as GA[2] does, but the performance of GlobalGA1 is clearly much better than that of GA[2], particularly in Case II-3 and Case II-4. This may suggest that the designs of the GlobalGA1 here, for example, the new NCP model, the new chromosome structure, and the associated operations, are more suitable for the NCP than the GA designs in [2].
- On average, GlobalGA2 achieves a better performance than GlobalGA1 does. Since GlobalGA2 has one more heuristic rule, that is, Rule 3, than GlobalGA1 has, it is reasonable to assume that the improvement in performance of GlobalGA2 is mainly due to Rule 3. As Rule 3 can also apply to GA[2], one may assume that the performance of GA[2] would also be improved if it employed Rule 3.
- It should be noted that, according to the fitness function given by (4) to (5) with *α*_1_ = *α*_2_ = 10, *α*_3_ = 1, *α*_4_ = 0, *α*_5_ = 200, and *α*_6_ = 0, the theoretical maximum fitness is 240 for Case II-1 to Case II-4.  Table 3 shows that GlobalGA3 always achieves this maximum fitness within 300 generations of evolution. From this table, one can see that GlobalGA3 converges much more quickly than GlobalGA1 and GlobalGA2, and it finds much better solutions than GlobalGA1 and GlobalGA2. Actually, GlobalGA3 always finds the theoretical optimal solutions. Since the only difference between GlobalGA3 and GlobalGA2 is the integration of Rules 4 and 5 into GlobalGA3, it is reasonable to conclude that it is the impact of these two additional rules that plays a significant role in improving the performance of the algorithm. It should be noted that these two rules, that is, Rules 4 and 5, are not designed only for the particular networks used in the experiments but developed without reference to any specific network topology, making them generally applicable regardless of topology.
- The theoretical optimal solutions in all cases require no coding, whilst Rule 3 initializes some chromosomes without coding. Therefore, could Rule 3 accidently introduce such theoretical optimal solutions into the gene pool right from the start, and then bias the GlobalGA2 and GlobalGA3 results? It should be pointed out that the no-coding solutions are not equal to the optimal solutions without coding. Actually, most no-coding solutions cannot achieve the theoretical maximum throughput. In other words, although the gene pool already includes some no-coding solutions due to Rule 3, it is very unlikely that such no-coding solutions can be guaranteed to be theoretical optimal solutions, and therefore they still need to evolve. For instance, Table 3 clearly shows that, on average, even GlobalGA3 needs to evolve tens of generations to find the theoretical optimal solutions. This implies that, most of the time, Rule 3 cannot introduce any theoretical optimal solution at all.

The above experimental results show that GlobalGA3 is the best algorithm, largely because of the introduction of Rules 4 and 5. Here we will further investigate the roles played by Rules 4 and 5. To save space, the experimental results reported here are all based on only one case, that is, Case II-4, which is the hardest case. In all previous experiments, when the focus was to improve a chromosome, GlobalGA3 applies Rules 4 and 5 no more than once. In other words, GlobalGA3 uses Rules 4 and 5 to modify no more than one gene of a chromosome. In the following experiments, we will allow GlobalGA3 to apply Rules 4 and 5 to modify up to *N*_R4R5_ genes of a chromosome, where *N*_R4R5_ = 1,…, 10. All other algorithm-related parameters remain the same as in previous experiments. The results are given in Table 4. From Table 4, the following observations can be made.

- GlobalGA3 can always find the theoretical optimal solutions, while GlobalGA1 and GlobalGA2 often struggle to do so. This proves that Rules 4 and 5 are the cause of the advantages.
- A GlobalGA3 with a larger *N*_R4R5_ needs fewer generations to converge to the optimal solutions. It is reasonable to suggest that Rules 4 and 5 play a crucial role in improving the performance of GlobalGA3: applying Rules 4 and 5 for more times will lead to better performance.
- However, applying Rules 4 and 5 causes additional computational burden; therefore, the computational time consumed by a generation of GlobalGA3 is larger than those of GlobalGA1 and GlobalGA2, and such computational time goes up as *N*_R4R5_ increases.
- Fortunately, when we combine the computational time consumed by a generation and the generations needed to converge to the optimal solutions, it becomes clear that the total computational time consumed by GlobalGA3 to find the optimal solutions is actually smaller than those of GlobalGA1 and GlobalGA2.
- Considering the influence of *N*_R4R5_ on the total computational time of GlobalGA3, a balance should be made to set up *N*_R4R5_, because the least total computational time occurs neither with a small *N*_R4R5_, nor with a large *N*_R4R5_, but with a medium *N*_R4R5_. In the case of Case II-4, the best value for *N*_R4R5_ is 8, which results in GlobalGA3 being able to find the optimal solutions at the fastest speed.

Hence it may be concluded that GlobalGA3 outperforms GlobalGA1 and GlobalGA2 in terms of not only solution quality, but also in terms of computational efficiency. This shows that the introduction of Rules 4 and 5 is very advantageous and hence justifies the use of the permutation representation.

As is well known, a large enough field size plays a crucial role in achieving the maximum possible throughput. Equation (13) shows that, in the case of our new GAs, the search space size for a single outgoing link will grow exponentially with the field size. Therefore, the focus of the following experiments is to explore and examine the influence of field size on the performance of our new GAs. Five field sizes, that is, *N*_*W*_ = 2, 4, 6, 8, and 10, are used in GlobalGA1, GlobalGA2, and GlobalGA3. Here *N*_R4R5_ is set as 8 for GlobalGA3, as Table 4 shows it gives the best performance. Based on those networks in Set II of Table 1, some key average results are given in Table 5, from which, the following observations can be made.

- The field size has a significant influence on the performance of GlobalGA1 and GlobalGA2. In the case of Case II-1, the simplest network of all, GlobalGA1 and GlobalGA2 with different field size can always find the optimal solutions, but it takes more time when a larger *N*_*W*_ is adopted. In the case of Case II-2 and Case II-3, GlobalGA1 and GlobalGA2 may still find the optimal solutions when *N*_*W*_ is small, but the solution quality reduces quickly as *N*_*W*_ increases. In Case II-4, the most complex network of all, both algorithms struggle and usually can only find feasible solutions, regardless of the value of *N*_*W*_.
- In all test cases, in terms of either solution quality or computational time, GlobalGA3 has a very robust performance against the change of *N*_*W*_. Actually, for a given network, GlobalGA3 can always find the optimal solution with similar computational time, no matter what value *N*_*W*_ has.
- In summary, one can see that the field size has significant influence on GlobalGA1 and GlobalGA2, which have relatively poor local-searching capability, but, thanks to Rules 4 and 5, no obvious influence on GlobalGA3 is observed. In other words, GlobalGA3 can perform satisfactorily well for different field sizes.

Based on the test cases for GlobalGA3 in Table 5, where *N*_*W*_ makes no difference in the performance of GlobalGA3, one may ask: how important is field size for exact network coding? In fact, the importance of field size is mainly appreciated in random network coding, because a larger field size means a higher probability of achieving the target rate when random coding is used. However, even for a small field size, say *N*_*W*_ = 2, there could still exist a coding scheme to achieve the target rate. For instance, in a rectangular grid network where the source sends out two signals using the random coding scheme, the probability that a node located at grid position (*x*, *y*) relative to the source can decode both signals is at least (1−1/*N*_*W*_)^2(*x*+*y*−2)^ [26]. This implies that, for all *N*_*W*_ ≥ 2, a rate of 2 is in theory always achievable at any node in a finite grid network. Unfortunately, the probability is so small under a small field size; say *N*_*W*_ = 2, that random coding can hardly determine a correct coding scheme. By employing a powerful method of searching, such as GlobalGA3 proposed in this paper, exact network coding may still stand a good chance of finding a correct coding scheme, even when *N*_*W*_ = 2. In other words, the field size might not be as important to exact network coding as is it to random network coding. This is definitely an issue worth further investigation in future research.

### 5.2. Tests on SRHCGA

**Figure 14:**
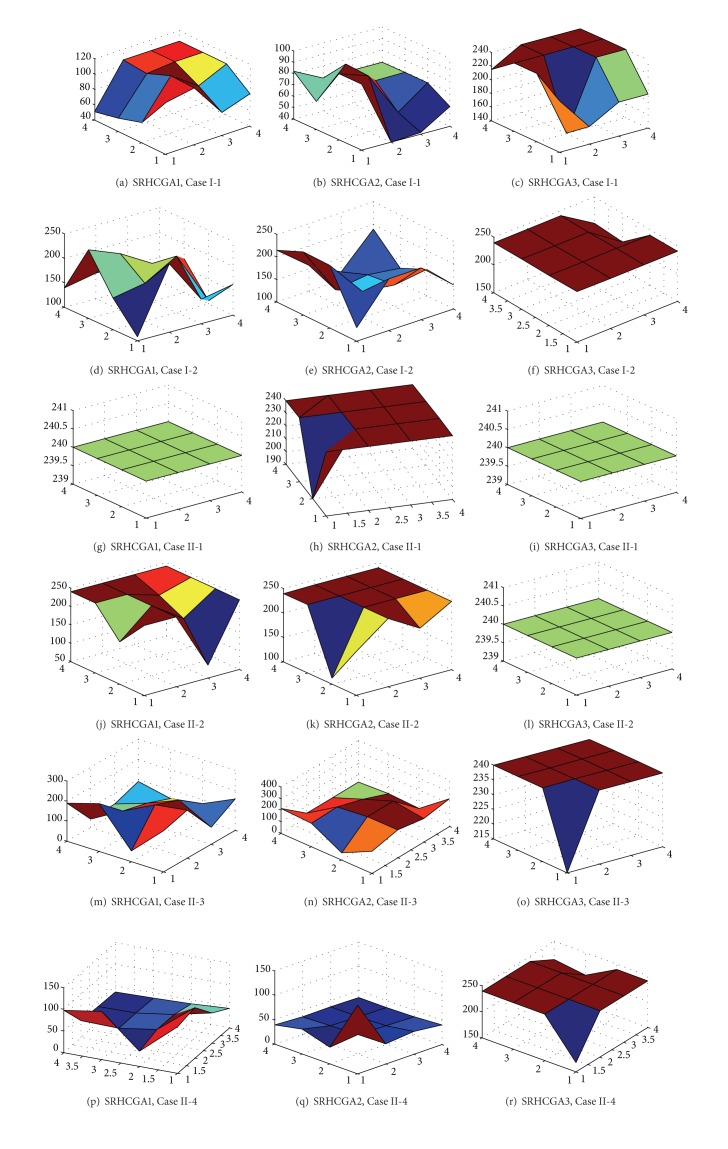
Influence of *N*_SL_ and *N*_*H*_ on fitness in general tests on SRHCGAs.

In this subsection, we will study the proposed SRHC strategy. We will firstly test the general performance of the SRHCGA. Then we will investigate the influence of some SRHC-related parameters. Like in the tests on the GlobalGA, here again we have three versions of the SRHCGA: SRHCGA1 employing Rules 1 and 2, SRHCGA2 having Rule 1 to Rule 3, and SRHCGA3 including Rule 1 to Rule 5 (Rules 4 and 5 are only applied no more than once; that is, *N*_R4R5_ = 1). In the general tests, for all three SRHCGAs, the number of steps in a spatial horizon, *N*_*H*_, changes from 1 to 4, and the spatial step length *N*_SL_ (i.e., how many link layers is covered by a spatial step) also changes from 1 to 4. Therefore, there are 16 (*N*_*H*_, *N*_SL_) pairs that are tested, and the total number of link layers covered by a spatial horizon varies from 1 to 16. The three SRHCGAs in the general tests share the same other SRHC-related parameters which are properly set up and fixed. For each pair of *N*_*H*_ and *N*_SL_, 20 tests are conducted for each SRHCGA. Some important average results and the associated values for *N*_*H*_ and *N*_SL_ are summarized in Table 6, and the relationships between *N*_*H*_, *N*_*SL*_, and the associated average fitness are plotted in Figure 14. The vertical axis in Figure 14 is the fitness axis, the first horizontal axis (the left horizontal axis) is the *N*_*H*_ axis, and the second horizontal axis (the right horizontal axis) is *N*_SL_ axis. It should be noted that, according to (8), that is, the unified objective function for the SRHC strategy, the potential maximum fitness is 240 when *β*_1_ = 20, *β*_2_ = 5, *β*_3_ = 5, *β*_4_ = 200, *β*_5_ = 0, and *β*_6_ = 10.

From Table 6 and Figure 14, one may have the following observations.

- From the typical (*N*_*H*_, *N*_SL_) pairs given in Table 6, one can see that small *N*_*H*_ and *N*_SL_ can deliver the best results in all test cases, which means it is not necessary to resolve the NCP as a whole, an appropriate problem partitioning method, such as the proposed SRHC strategy, can eventually find at least as good complete solutions to the NCP as the “resolve it as a whole” strategy can.
- Actually, when comparing the average minimal coding links and the minimal actually achieved rate at sinks in Tables 3 and 6, one can see that in Case I-1, Case I-2, Case II-3, and Case II-4, which have relatively larger network scale, the performances of SRHCGA1 and SRHCGA2 with small *N*_*H*_ and *N*_SL_ are obviously better than those of GlobalGA1 and GlobalGA2. As discussed in the previous tests on GlobalGAs, GlobalGA1 and GlobalGA2 have relatively poorer searching capability because they do not use Rules 4 and 5. When such an algorithm is applied to resolve a relatively larger NCP as a whole, it is difficult to find good solutions. However, when such an algorithm with poor searching capability is integrated with the proposed SRHC strategy, its performance can be improved.
- Regarding the average minimal coding links and the minimal actually achieved rate at sinks, SRHCGA3 does not make difference when compared with GlobalGA3. This is because, thanks to Rules 4 and 5, GlobalGA3 is so powerful that it can almost always find the optimal solutions. Therefore, there is no room for SRHCGA3 to improve.
- Based on the above three bullet points, one may conclude that, for a given algorithm, (i) if the “resolve it as a whole” strategy can find the best solutions, then the SRHC strategy can also do it, and (ii) if the “resolve it as a whole” strategy struggles in finding the best solutions, the SRHC strategy may still find the best solutions, or at least find some better solutions.
- When comparing the total computational times in Case II-4 consumed by SRHCGA1 to SRHCGA3 in Case II-4 and those by GlobalGA1 to GlobalGA3 with *N*_R4R5_ = 1 (see Table 4), one can see clearly that SRHCGA1 to SRHCGA3 are much more time-efficient than GlobalGA1 to GlobalGA3. This is understandable. The computational time of GAs usually soars up exponentially as the problem scale increases. The computational time consumed by an SRHCGA within a spatial horizon is therefore exponentially less than that by a GlobalGA (because the subproblem within a spatial horizon has a smaller problem scale). Even though an SRHCGA needs to experience a number of spatial horizons in order to get a complete solution, the total computational time just increases linearly and therefore is still less than that of a GlobalGA. Now it is clear that, for the NCP, the SRHC strategy is advantageous against the “resolve it as a whole” strategy in terms of both solution quality and computational time.
- Figure 14 reveals more details regarding how the SRHCGAs perform with different (*N*_*H*_, *N*_SL_) pairs. Basically, the largest fitness is achieved or can be achieved under a small (*N*_*H*_, *N*_SL_) pair in all tests cases. In particular, when an algorithm with poor searching capability, such as SRHCGA1 and SRHCGA2, is applied to a large-scale NCP, the largest fitness is always achieved under small (*N*_*H*_, *N*_SL_) pairs, and larger (*N*_*H*_,  *N*_SL_) pairs usually have very small fitness (see Figures 14(a), 14(b), 14(d), 14(e), 14(m), 14(n), and 14(p)). These are in line with the observations made based on Table 6 and therefore further prove that “resolve it as a whole” strategy is not necessary and sometimes even disadvantageous.
- From Figure 14, one may notice that the smallest (*N*_*H*_, *N*_SL_) pair, that is, *N*_*H*_ = 1 and *N*_SL_ = 1, usually does not give the largest fitness. Even for SRHCGA3, the best algorithm of all, *N*_*H*_ = 1 and *N*_SL_ = 1 may lead to nonoptimal solutions; for example, see Figures 14(c), 14(o), and 14(r). One may also notice in Figure 14 that even SRHCGA3 fails to achieve the largest fitness sometimes when *N*_*H*_ and *N*_SL_ are both large; for example, see Figures 14(f) and 14(r). This may imply that it is crucial to find a suitable length for spatial receding horizon. A too small spatial horizon may lead to shortsighted performance, whilst a too large spatial horizon will likely make the SRHCGA strategy similar to the “resolve it as a whole” strategy. Therefore, a balance should be made when setting up the (*N*_*H*_, *N*_SL_) pair. According to Table 6 and Figure 14, it seems that (2,1), (2,2), and (3,1) are good (*N*_*H*_, *N*_SL_) pairs for the NCP. One may ask: given a really large network that has thousands of link layers, will these three (*N*_*H*_, *N*_SL_) pairs, which now appear very small, still be able to deliver good rather than shortsighted performance? This question may be partially answered in the following tests on the importance of the terminal penalty term in the objective function of SRHCGA.

It is well known in the area of control engineering that the TRHC scheme may become unstable if no terminal penalty is included in the objective function. Simply speaking, a terminal penalty term is used to estimate the impact of the decisions made within the current temporal horizon on the future system behavior. Similarly, the terminal penalty term introduced for the SRHC strategy in Section 3.2 is used to estimate the impact of the decisions made within the current spatial horizon on those undecided links that are beyond the current spatial horizon.  Table 7 compares the performances of SRHCGAs before and after the terminal penalty term is removed from the objective function. Only the typical (*N*_*H*_, *N*_*SL*_) pairs given in Table 6, which have delivered the best average results in the associated test cases, are used to conduct the new experiments associated with Table 7. To save space, only the results of average best fitness are given in Table 7. One can see clearly from Table 7 that, once the terminal penal is removed from the objective function, the performances of all SRHCGAs degrade dramatically in almost all test cases. Even SRHCGA3 often fails to find optimal solutions, even in the simplest test case, that is, Case II-1. This clearly verifies the importance of terminal penalty for the SRHC strategy in the NCP. The reason for the crucial role of terminal penalty was already explained in Section 3.2 (see Figure 9). From the importance of terminal penalty along with Rules 4 and 5, one may see a nature of the NCP: if the signals received by a link layer are better organized and diversified, then it is more likely that a node of the following link layer can receive more different signals. In other words, focusing on organizing and diversifying local information flow (to some extent the SRHC strategy does this job) may also lead to high-quality coding solutions for the entire network. This nature of the NCP may somehow explain why small (*N*_*H*_, *N*_SL_) pairs can always get the best results in all test cases in Table 6 and Figure 14. According to this nature, some small (*N*_*H*_, *N*_SL_) pairs, such as those revealed in Table 6 and Figure 14, might still be able to give satisfactory performance even in a network with thousands of link layers. This is because, as long as the signals on each link layer are well organized, the sink layer will receive a reasonably large number of diversified signals.

As discussed in Section 2.3, the SRHC strategy and population-based algorithms like GAs are a perfect match. One reason for this perfect match is that a GA will output a population of solutions, some of which can be then used to set up a pool for decided spatial steps, in order to avoid generating bad complete solutions caused by the uniqueness of subsolutions for decided spatial steps. In the experiments given as follows, we will test whether or not such a pool for decided spatial steps is useful. In all previous SRHCGA related experiments, the pool size was set as 10% of a GA population. Here another three pool sizes are used: 1 candidate combination only, 5% of a GA population, and 20% of a GA population. The results on average best fitness are listed in Table 8, from which one can see clearly that, in general, a smaller pool size will lead to a poorer performance for all SRHCGAs. As analyzed in Section 2.3, a smaller pool size means less flexibility in changing the subsolutions for decided links, so, an algorithm is more likely to be trapped to locally good solutions. If the pool only has one candidate, then it makes no difference in terms of flexibility for decided links when the SRHC strategy uses either a population-based algorithm or a deterministic algorithm, and the resulting performance is very poor (even SRHCGA3 often fails to find optimal solutions, even in the simplest test case, i.e., Case II-1). Table 8 also reveals that a too large pool size is not necessary, as it will not improve the performance further or significantly. Actually, a too large pool size may unnecessarily increase the complexity of overall search space, and consequently the performance of an algorithm with poor searching capability will degrade (see SRHCGA1 and SRHCGA2 in Table 8, e.g.). In the NCP experiments, a pool size that is 10% of a GA population seems able to give reasonably good performance for all SRHCGAs. Now one may conclude that a subsolution pool for decided spatial steps plays a crucial role for the SRHC strategy to achieve good performance. Therefore, a deterministic algorithm, which only outputs a single solution, is not suitable for conducting the optimization within a spatial receding horizon. Instead, only a population-based algorithm like GA can take the full advantage of the SRHC strategy.

## 6. Conclusions

This paper attempts to develop an effective genetic algorithm (GA) for the network coding problem (NCP), where network coding resources such as coding nodes and links need to be minimized. The contributions of this reported work include the following. (i) A new mathematical formulation of the NCP is developed, which aims not only to minimize network coding resources, but also to maximize the actually achieved rate at sinks. (ii) A novel permutation representation instead of widely used binary matrix is proposed, which records relative signals on links and is therefore free of feasibility problems, and which also enables the derivation of exact information flow on links and consequently makes it possible to integrate many useful problem-specific knowledge into the algorithm. (iii) Some new NCP-specific heuristic rules are reported, which can significantly improve the overall quality of chromosomes. (iv) A novel spatial receding horizon control (SRHC) strategy is invented as problem partitioning method, which is very effective to decompose large-scale networks and is also suitable for population-based algorithms, such as GAs, and therefore makes the proposed SRHC based GA have a good scalability for the NCP. The effectiveness of these new developments is illustrated by extensive experiments. It is worth investigation to generalize the SRHC scheme, in order to develop a general problem partitioning methodology of combining SRHC with population-based algorithms to apply to various large-scale problems.

## Figures and Tables

**Table 1:** Networks used in different test cases.

	Copy the network Figure 1(b) or generated by [28]	Nodes	Links	Sinks	Target rate
Case I-1	Generated by [28]	20	80	12	4
Case I-2	Generated by [28]	40	120	12	3

Case II-1	3 copies of Figure 1(b)	19	30	4	2
Case II-2	7 copies of Figure 1(b)	43	70	8	2
Case II-3	15 copies of Figure 1(b)	91	150	16	2
Case II-4	31 copies of Figure 1(b)	187	300	32	2

**Table 2:** Comparative results with existing methods (number of coding links).

	Case I-1		Case I-2		Case II-1		Case II-2		Case II-3		Case II-4	
	Best	Ave.	Best	Ave.	Best	Ave.	Best	Ave.	Best	Ave.	Best	Ave.
Minimal 1	0	1.35	0	1.85	3	3.00	7	7.00	15	15.00	31	31.00
Minimal 2	0	1.85	0	1.90	0	2.15	2	4.70	7	11.60	28	52.80
GA [2]	0	1.20	0	1.05	0	0.65	0	2.15	3	5.35	12	17.20
GlobalGA1	0	1.20	0	0.80	0	0.00	0	0.00	0	0.80	0	6.30
GlobalGA2	0	1.15	0	0.70	0	0.00	0	0.00	0	0.30	0	5.00
**GlobalGA3**	**0**	**0.00**	**0**	**0.00**	**0**	**0.00**	**0**	**0.00**	**0**	**0.00**	**0**	**0.00**

**Table 3:** Details of the results of the new GlobalGAs.

(Average results of 20 runs)	Case I-1	Case I-2	Case II-1	Case II-2	Case II-3	Case II-4
Final max fitness						
GlobalGA1	149.65	190.00	240.00	230.00	181.67	40.21
GlobalGA2	171.54	195.66	240.00	240.00	195.64	59.09
**GlobalGA3**	**280.00**	**260.00**	**240.00**	**240.00**	**240.00**	**240.00**

How many generations to achieve final max fitness						
GlobalGA1	245.50	239.60	2.35	9.40	242.40	300.00
GlobalGA2	210.75	221.00	1.05	5.80	171.90	300.00
**GlobalGA3**	**64.40**	**39.10**	**1.00**	**2.20**	**11.45**	**56.70**

Average minimal coding links						
GlobalGA1	1.20	0.80	0.00	0.00	0.80	6.30
GlobalGA2	1.15	0.70	0.00	0.00	0.30	5.00
**GlobalGA3**	**0.00**	**0.00**	**0.00**	**0.00**	**0.00**	**0.00**

Maximum minimal coding links						
GlobalGA1	4	3	0	1	3	22
GlobalGA2	3	1	0	0	2	12
**GlobalGA3**	**0**	**0**	**0**	**0**	**0**	**0**

Minimum actually achieved rate at sinks						
GlobalGA1	3	3	2	2	1	1
GlobalGA2	3	3	2	2	2	1
**GlobalGA3**	**4**	**3**	**2**	**2**	**2**	**2**

**Table 4:** Computational efficiencies of different GlobalGAs based on Case II-4.

(Ave. results of 20 exp.)	GlobalGA1	GlobalGA2	GlobalGA3 with a *N*_R4R5_ of									
			1	2	3	4	5	6	7	8	9	10
Final max fitness	40.21	59.09	**240.00**	**240.00**	**240.00**	**240.00**	**240.00**	**240.00**	**240.00**	**240.00**	**240.00**	**240.00**
Number of coding links	6.30	5.00	**0.00**	**0.00**	**0.00**	**0.00**	**0.00**	**0.00**	**0.00**	**0.00**	**0.00**	**0.00**
Generations to converge	300.00	300.00	56.70	28.40	15.60	11.10	4.30	3.90	3.40	3.00	3.00	**2.70**
Computational time of one generation (sec.)	1.39	**1.38**	2.92	3.42	4.16	4.85	5.78	6.48	7.09	7.28	7.82	8.72
Total computational time (sec.)	415.70	414.57	168.31	96.78	60.87	49.91	24.91	25.36	24.18	**22.00**	23.45	23.83

**Table 5:** The influence of field size on the performance of new GAs.

(Ave. results of 20 exp.)	*N*_*W*_				
	2	4	6	8	10
GlobalGA1					
Case II-1					
Final max fitness	240.00	240.00	240.00	240.00	240.00
Total computational time (sec.)	0.48	0.81	1.06	3.44	5.56
Case II-2					
Final max fitness	240.00	240.00	123.33	115.68	90.61
Total computational time (sec.)	2.40	59.26	88.65	93.46	89.27
Case II-3					
Final max fitness	181.67	54.09	50.05	49.72	52.18
Total computational time (sec.)	158.85	200.61	201.12	200.25	200.42
Case II-4					
Final max fitness	40.21	45.33	42.95	39.76	41.03
Total computational time (sec.)	415.70	420.58	405.17	411.54	425.67

GlobalGA2					
Case II-1					
Final max fitness	240.00	240.00	240.00	240.00	240.00
Total computational time (sec.)	0.47	0.47	0.50	0.48	0.47
Case II-2					
Final max fitness	240.00	240.00	240.00	240.00	240.00
Total computational time (sec.)	2.76	6.88	7.85	6.55	8.43
Case II-3					
Final max fitness	195.64	142.86	45.96	47.45	46.11
Total computational time (sec.)	127.24	201.60	199.53	200.97	204.34
Case II-4					
Final max fitness	59.09	40.16	39.50	41.88	42.02
Total computational time (sec.)	414.57	429.06	418.63	424.41	419.24

GlobalGA3					
Case II-1					
Final max fitness	240.00	240.00	240.00	240.00	240.00
Total computational time (sec.)	2.12	2.05	1.97	2.16	2.01
Case II-2					
Final max fitness	240.00	240.00	240.00	240.00	240.00
Total computational time (sec.)	3.37	4.15	3.68	3.44	3.51
Case II-3					
Final max fitness	240.00	240.00	240.00	240.00	240.00
Total computational time (sec.)	5.36	5.72	6.44	5.51	7.70
Case II-4					
Final max fitness	240.00	240.00	240.00	240.00	240.00
Total computational time (sec.)	22.00	19.24	21.27	24.84	19.13

**Table 6:** Summarized results of general tests on SRHCGAs.

(Best average results of all combinations of *N*_SL_ and *N*_*H*_)	Case I-1	Case I-2	Case II-1	Case II-2	Case II-3	Case II-4
Average minimal coding links						
SRHCGA1	0.75	0.00	0.00	0.00	0.00	1.50
SRHCGA2	0.75	0.00	0.00	0.00	0.00	0
**SRHCGA3**	**0.00**	**0.00**	**0.00**	**0.00**	**0.00**	**0.00**

Total computational time (sec.)						
SRHCGA1	35.61	11.94	0.00	4.72	12.89	64.59
SRHCGA2	28.25	9.83	0.00	3.35	11.17	58.93
SRHCGA3	32.86	6.04	0.00	3.82	8.43	27.38

Minimum actually achieved rate at sinks						
SRHCGA1	3.5	3	2	2	2	1.75
SRHCGA2	3.75	3	2	2	2	1.50
**SRHCGA3**	**4**	**3**	**2**	**2**	**2**	**2**

A typical (*N*_*H*_, *N*_SL_) pair that gives the best average results						
SRHCGA1	(2,2)	(3,1)	(2,2)	(2,2)	(3,1)	(1,2)
SRHCGA2	(2,1)	(3,1)	(2,2)	(2,2)	(2,2)	(1,1)
SRHCGA3	(2,1)	(2,1)	(2,2)	(2,2)	(2,2)	(2,2)

How many (*N*_*H*_, *N*_SL_) pairs that give the best average results						
SRHCGA1	1	2	16	12	2	1
SRHCGA2	2	2	16	13	4	1
SRHCGA3	10	14	16	16	16	13

**Table 7:** The importance of terminal penalty in objective function for SRHCGAs.

(Based on the typical (*N*_*H*_, *N*_SL_) pairs given in Table 6)	Case I-1	Case I-2	Case II-1	Case II-2	Case II-3	Case II-4
SRHCGA1						
With terminal penalty	**115.10**	**240.00**	**240.00**	**240.00**	**240.00**	**116.94**
Without terminal penalty	69.47	165.77	220.35	203.71	104.68	32.07

SRHCGA2						
With terminal penalty	**98.17**	**240.00**	**240.00**	**240.00**	**240.00**	**140.07**
Without terminal penalty	47.29	144.75	228.60	185.95	123.66	40.51

SRHCGA3						
With terminal penalty	**240.00**	**240.00**	**240.00**	**240.00**	**240.00**	**240.00**
Without terminal penalty	159.55	172.81	231.92	222.90	195.85	147.62

**Table 8:** The influence of pool size on performance of SRHCGAs.

(Based on the typical (*N*_*H*_, *N*_SL_) pairs given in Table 6)	Pool size	Case I-1	Case I-2	Case II-1	Case II-2	Case II-3	Case II-4
SRHCGA1	1	40.53	116.38	217.44	179.36	122.97	43.47
	5% a GA population	110.47	214.61	**240.00**	**240.00**	221.85	**122.73**
	10% a GA population	**115.10**	**240.00**	**240.00**	**240.00**	**240.00**	116.94
	20% a GA population	101.26	225.36	**240.00**	233.85	217.81	98.48

SRHCGA2	1	49.38	100.94	225.63	194.95	151.38	51.55
	5% a GA population	76.86	230.44	**240.00**	236.04	213.25	118.87
	10% a GA population	98.17	**240.00**	**240.00**	**240.00**	**240.00**	**140.07**
	20% a GA population	**108.47**	226.75	**240.00**	**240.00**	**240.00**	133.79

SRHCGA3	1	167.53	182.44	232.35	235.11	194.69	151.36
	5% a GA population	233.85	**240.00**	**240.00**	**240.00**	**240.00**	229.74
	10% a GA population	**240.00**	**240.00**	**240.00**	**240.00**	**240.00**	**240.00**
	20% a GA population	**240.00**	**240.00**	**240.00**	**240.00**	**240.00**	**240.00**

## References

[1] Ahlswede R, Cai N, Li S-YR, Yeung RW (2000). Network information flow. *IEEE Transactions on Information Theory*.

[2] Kim M, Ahn CW, Medard M, Effros M On minimizing network coding resources: an evolutionary approach.

[3] Richey MB, Parker RG (1986). On multiple steiner subgraph problems. *Networks*.

[4] Fragouli C, Parker RG (2006). Information flow decomposition for network coding. *IEEE Transactions on Information Theory*.

[5] Langberg M, Sprintson A, Bruck J (2006). The encoding complexity of network coding. *IEEE Transactions on Information Theory*.

[6] Bhattad K, Ratnakar N, Koetter R, Narayanan KR Minimal network coding for multicast.

[7] Kim M, Médard M, Aggarwal V Evolutionary approaches to minimizing network coding resources.

[8] Kim M, Aggarwal V, O’Reilly UM, Medard M, Kim W Genetic representation for evolutionary minimization of network coding resources.

[9] Kim M, Aggarwal V, O’Reilly U-M, Medard M A doubly distributed genetic algorithm for network coding.

[10] Hu X-B, Leeson MS, Hines EL (2012). An effective genetic algorithm for network coding. *Computers and Operations Research*.

[11] Thierens D (1999). Scalability problems of simple genetic algorithms. *Evolutionary computation*.

[12] Cantú-Paz E, Goldberg DE (1999). On the scalability of parallel genetic algorithms. *Evolutionary computation*.

[13] Colombo G, Allen SM Problem decomposition for minimum interference frequency assignment.

[14] Tsutsui S, Ghosh A, Fujimoto Y (1997). Forking genetic algorithms: GAs with search space division schemes. *Evolutionary Computation*.

[15] Clarke DW (1994). *Advances in Model-Based Predictive Control*.

[16] Maciejowski JM (2002). *Predictive Control with Constraints*.

[17] Hu X-B, di Paolo EA (2011). A ripple-spreading genetic algorithm for the aircraft sequencing problem. *Evolutionary Computation*.

[18] Chand S, Hsu VN, Sethi S (2002). Forecast, solution, and rolling horizons in operations management problems: a classified bibliography. *Manufacturing and Service Operations Management*.

[19] De Schutter B, Van Den Boom T (2001). Model predictive control for max-plus-linear discrete event systems. *Automatica*.

[20] Hu X-B, Chen W-H (2005). Genetic algorithm based on receding horizon control for arrival sequencing and scheduling. *Engineering Applications of Artificial Intelligence*.

[21] Hu X-B, Chen W-H, Di Paolo E (2007). Multiairport capacity management: genetic algorithm with receding horizon. *IEEE Transactions on Intelligent Transportation Systems*.

[22] Zhan Z-H, Zhang J, Li Y (2010). An efficient ant colony system based on receding horizon control for the aircraft arrival sequencing and scheduling problem. *IEEE Transactions on Intelligent Transportation Systems*.

[23] Fonseca CM, Fleming PJ (1995). An overview of evolutionary algorithms in multiobjective optimization. *Evolutionary Computation*.

[24] Eiben AE, Smith JE (2003). *Introduction to Evolutionary Computing*.

[25] Ho T, Medard M, Shi J, Effros M, Karger DR On randomized network coding.

[26] Ho T, Koetter R, Médard M, Karger DR, Effros M The benefits of coding over routing in a randomized setting.

[27] Sywerda G (1989). Uniform crossover in genetic algorithms.

[28] Melançon G, Philippe F (2004). Generating connected acyclic digraphs uniformly at random. *Information Processing Letters*.

